# Demographic and prognostic factors of optic nerve astrocytoma: a retrospective study of surveillance, epidemiology, and end results (SEER)

**DOI:** 10.1186/s12885-021-08719-2

**Published:** 2021-08-30

**Authors:** Mingui Zhang, Tao Chen, Yisheng Zhong

**Affiliations:** 1 Department of Ophthalmology, Ruijin Hospital Affiliated Medical School, Shanghai Jiaotong University, 197 Ruijin Er Road, Shanghai, 200025 China; 2 Department of Ophthalmology, Massachusetts Eye and Ear Infirmary / Schepens Eye Research Institute, Harvard University, Boston, MA 02114 USA; 3 Department of Thoracic Surgery, Shanghai Pulmonary Hospital, Shanghai, China

**Keywords:** SEER, Optic nerve, Astrocytoma, Survival, Age

## Abstract

**Background:**

Optic nerve astrocytomas (ONAs) are neurological neoplasms in the central nervous system (CNS), and they have the highest incidence rate among all the tumor types in the visual pathway. In this study, we conducted a Surveillance, Epidemiology, and End Results (SEER) -based research to explore the demographic, survival, and prognostic factors of patients diagnosed with ONAs.

**Methods:**

Utilizing the SEER database, we retrospectively evaluated data of patients diagnosed with ONAs of all ages from 1984 to 2016. We used the Student’s t distribution to test variables of patients and various characteristics, and Kaplan-Meier curve to illustrate overall survival (OS) with 95.0% confidence intervals (CIs). We also performed univariate and multivariate analyses to evaluate various variables’ validity on overall survival.

**Results:**

A total of 1004 cases were analyzed, and revealed that age (P<0.001, hazard ratio (HR) = 8.830, 95% CI: 4.088–19.073), tumor grade (P<0.001, HR = 1.927, 95% CI: 1.516–2.450), diagnostic confirmation (P<0.001, HR = 2.444, 95% CI: 1.632–3.660), and histology type (*P* = 0.046, HR = 1.563, 95% CI: 1.008–2.424) of the tumor were associated with decreased survival.

**Conclusions:**

From this large, comparative study of ONAs, we found that younger age may be considered as a protective indicator, while high-grade astrocytic tumors have a worse prognosis. We also found that diagnostic confirmation and tumor grade were independent prognostic factors in this patient population.

## Background

In the central nervous system (CNS), there are three types of tumors: astrocytoma, oligodendroglioma, and ependymoma. Of all these tumors, astrocytoma is the most common type [1–3]. ONAs are rare astrocytic tumors that occur in the optic nerve and reach out to the chiasm and the frontal lobe frequently [4, 5]. The classification of ONAs is based upon the World Health Organization (WHO) criteria, and it include Grade I (pilocytic), Grade II (diffuse), Grade III (anaplastic), and Grade IV (glioblastoma) astrocytoma [6]. Pilocytic astrocytoma has the highest incidence rate in people and it has excellent prognosis and survival rate [3, 7–10].

As 50–60% of patients with ONAs have neurofibromatosis type 1 (NF-1), the mutation in the NF-1 suppressor gene is considered to be a predictor for developing ONAs [2, 11–14]. Most patients are in the pediatric population between the ages of 0 and 14 years [15]. The 5-year survival rate of optic nerve astrocytoma is over 95.0% [16], while more than 3 quarters of patients’ vision is greatly impaired [14, 17, 18].

For a long period, ONAs were regarded as indolent diseases and did not require therapy [19]. However, the latest research suggests that ONAs have an unpredictable clinical process, ranging from rapid progression [20, 21] to spontaneous regression [22]. Some physicians had a preference for surgical treatment [23], while some tended to utilize radiotherapy treatment for ONAs patients [24]. Recently, chemotherapy and observation is believed to be an effective therapeutic method for ONAs [25, 26]. ONAs have unpredictable progression, and the consequences are highly associated with treatment modalities, thus these facts lead to ONAs’ controversial treatment choices [27].

During last decades, only a few studies had focused on the characteristics and outcomes in optic pathway gliomas in the US, but none have focused on the incidence and outcomes of ONAs in population. Sustaining observation of this disease on population is crucial. On one hand, it can help researchers evaluate the efficiency of medical care, on the other hand, it can help improves physicians’ comprehension of ONAs [28, 29]. To better understand the epidemiology, age at diagnosis, racial and sex differences, histology type and diagnostic confirmation of ONAs in the US, we conducted a retrospective demographic study using the SEER database of the National Cancer Institute. SEER is an authoritative source for cancer statistics in the United States. The SEER Program provides information on cancer statistics in an effort to reduce the cancer burden among the U.S. population.

## Materials and methods

### Patient selection and data collection

All data were extracted from the latest SEER database with SEER*Stat software: SEER 21 (Nov 2019 release) [14]. The SEER registry provides comprehensive cancer data including incidence and survival rates which obtained from medical records, covering up approximately 30.0% of the US population [14, 30]. Data from 1984 to 2016 and a total of 1004 patients with ONAs were extracted from the SEER database. To increase the accuracy of the research, we use codes “C72.3-Optic nerve” to identify patients with optic nerve cataloged as the original tumor site [31]. Patients with ONAs were identified based on International Classification of Disease for Oncology, 3rd edition (ICD-O-3) histology codes for astrocytoma (9380, 9400–9421). Patients had another primary malignancy were excluded from our study.

To analyse data, we converted continuous variables to categorical variables. We extracted data on demographic and clinical variables, including age (0–18 years, 18+ years), race [White, Black, Asian, Other (American Indian /Alaska Native /Pacific Islander), Unknown], sex (male, female), tumor grade, year of diagnosis, and survival months until death or follow-up as of December thirty-first, 2016. The study followed the SEER database manual to conduct procedures performed on patients diagnosed with ONAs [31]. In our study, surgery performances of patients were classified into 3 categories: No surgery performance; surgery not otherwise specified (Surgery, NOS), and surgery status unknown (SSU). Histology types were classified as follows: low-grade astrocytic tumors, high-grade astrocytic tumors, and astrocytoma not otherwise specified (NOS). Pilocytic astrocytoma, diffuse astrocytoma, anaplastic astrocytoma, and glioblastoma were considered as grade I, II, III, IV respectively. Grade I and grade II were deemed as low-grade astrocytic tumors, while grade III and grade IV were deemed as high-grade astrocytic tumors. The data includes both malignant and non-malignant tumors.

Patients without complete survival information were deleted from this research. ONAs-specific survival was survival related with ONAs.

### Statistical analyses

All statistical computations in this research were conducted utilizing the IBM Statistical Package for Social Science (SPSS, Inc., Chicago, IL) Statistics software, version 20 for Mac. The Student’s *t* test or *Pearson* correlation test was applied to analyze quantitative variables in Table 1. Overall survival (OS) was calculated using Kaplan–Meier curves with 95% confidence intervals (CIs). If the risk factors were confirmed by univariate study, they were then adopted in multivariate Cox proportional hazard analysis. The correlation coefficient (r) is the measure of degree of interrelationship between variables. A *P* value of < 0.05 was considered statistically significant.

**Table 1:** Demographic and clinical characteristics of optic nerve astrocytoma cases (*n* = 1004)

Characteristic	No.	% of Total
**Age at diagnosis (years)**		
≤ 18	838	83.5
>18	166	16.5
**Sex**		
Female	520	51.8
Male	484	48.2
**Race**		
White	833	83.0
Black	80	8.0
Asian	44	4.4
Other	22	2.2
Unknown	25	2.5
**Year of diagnosis**		
<2010	616	61.4
≥ 2010	388	38.6
**Surgery performance**		
Surgery, NOS	169	16.8
No surgery	823	82.0
Unknown	12	1.2
**Tumor size**		
≤ 2	134	13.3
>2	106	10.6
Unknown	764	76.1
**Diagnostic Confirmation**		
Radiography without microscopic confirm	674	67.1
Clinical diagnosis only	25	2.5
Positive histology	281	28.0
Positive laboratory test/marker study	1	0.1
Direct visualization without microscopic confirmation	6	0.6
Positive microscopic confirm, method not specified	2	0.2
Unknown	15	1.5
**Tumor Extension**		
Unknown	171	17
Distant site(s)/node(s) involved	31	3.1
Localized only	713	71.0
Regional, NOS	89	8.9
**Laterality**		
Right - origin of primary	279	27.8
Left - origin of primary	244	24.3
Bilateral, single primary/paired site	244	24.3
Unknown	237	23.6
**Histology Type**		
Low-grade astrocytic tumors	953	94.9
High-grade astrocytic tumors	11	1.1
Astrocytoma, NOS	40	4

## Results

### Demographic characteristics

In this population-based study, a total of 1004 optic nerve astrocytoma patients who were diagnosed between 1984 and 2016 were enrolled. There were 484 (48.2%) male patients and 838 (83.5%) patients who were younger than 18 years old. The average age at diagnosis is 10.9 years old. Most of the patients were white (83.0%), while 8.0% were black and 4.4% were Asian. In this cohort, there were 953 (94.9%) tumors categorized as low-grade astrocytic tumors and 40 (4.0%) astrocytomas described as type NOS. Pilocytic astrocytoma was the most common histologic type with available data. Laterality information was reported for 76.4% of the patients: 27.8% of patients had a right origin of primary tumor, 24.3% had a left origin of primary tumor, and 24.3% had bilateral tumor involvement. Among all the patients, 38.6% were diagnosed in the period of 2010–2016. The majority of patients (82.0%) did not receive surgery. The demographic characteristics were summarized in Table 1.

There were significant statistical difference between age at diagnosis and sex (*r* = 0.070, *P* = 0.026), surgery performance (*r* = 0.064, *P* = 0.045), tumor size (*r* = 0.137, *P* = 0.034), tumor grade (*r* = 0.146, *P* < 0.001), diagnostic confirmation (*r* = 0.099, *P* = 0.002), laterality (*r* = 0.157, *P* < 0.001), and histology type (*r* = 0.168, *P* < 0.001)(Table 2). Race, year of diagnosis, and tumor extension did not result in significantly difference with age (*P* = 0.075, *P* = 0.950, *P* = 0.733, respectively). Amongst patients with known tumor grade, 96.7% of patients younger than 18 years old at diagnosis had low-grade astrocytic tumors (*n* = 810/838); 86.1% of patients who are older than 18 years old had a low-grade astrocytic tumor (*n* = 143/166).

**Table 2:** The Relationship between Age at Diagnosis and Clinical Characteristics in ONAs Patients

Variables	Age ≤ 18	Age>18	Correlation Coefficient	P Value
**Sex**			0.07	0.026
Female	425	95		
Male	413	71		
**Race**			0.057	0.075
White	704	129		
Black	59	21		
Asian	35	9		
Other	19	3		
Unknown	21	4		
**Year of diagnosis**			−0.002	0.950
<2010	504	112		
≥ 2010	334	54		
**Surgery performance**			0.064	0.045
Surgery, NOS	127	42		
No surgery	701	122		
Unknown	10	2		
**Tumor size**			−0.137	0.034
≤ 2	98	36		
>2	87	19		
Unknown	653	111		
**Tumor grade**			0.146	<0.001
I	30	12		
II	30	10		
III	1	1		
IV	5	16		
Unknown	772	127		
**Diagnostic Confirmation**			0.099	0.002
Radiography without microscopic confirm	588	86		
Clinical diagnosis only	21	4		
Positive histology	208	73		
Positive laboratory test/marker study	1	0		
Direct visualization without microscopic confirmation	6	0		
Positive microscopic confirm	2	0		
Unknown	12	3		
**Tumor Extension**			−0.011	0.733
Distant site(s)/node(s) involved	30	1		
Localized only	596	117		
Regional, NOS	76	13		
Unknown	136	35		
**Laterality**			−0.157	<0.001
Right - origin of primary	214	65		
Left - origin of primary	205	39		
Bilateral, single primary/paired site	221	23		
Unknown	198	39		
**Histology Type**			0.168	<0.001
Low-grade astrocytic tumors	810	143		
High-grade astrocytic tumors	1	10		
Astrocytoma, NOS	27	13		

### Survival and prognostic factors of optic nerve astrocytoma

**Fig. 1:**
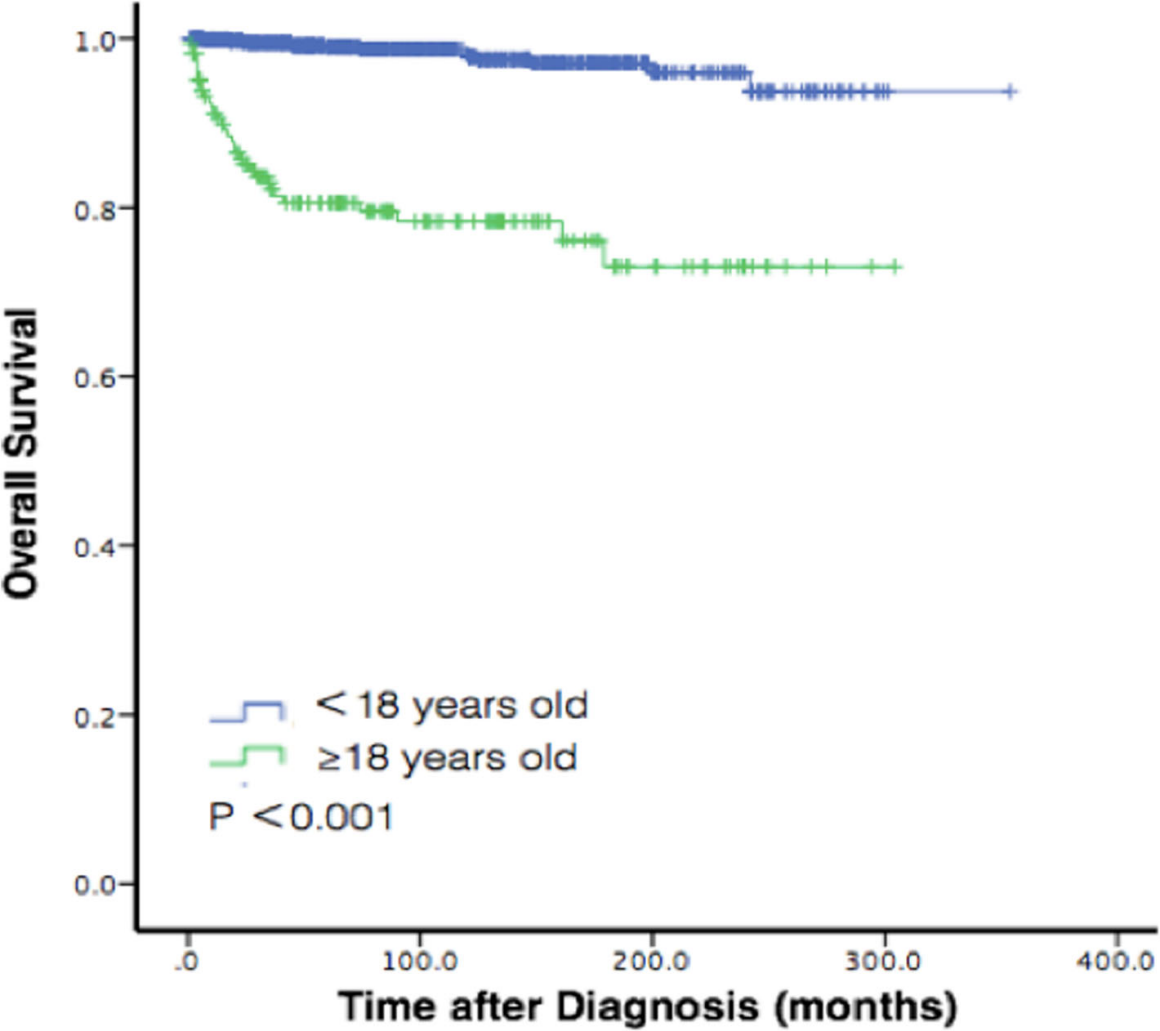
Kaplan-Meier survival analysis for overall survival by age groups 0–18 and 18+ years

**Fig. 2:**
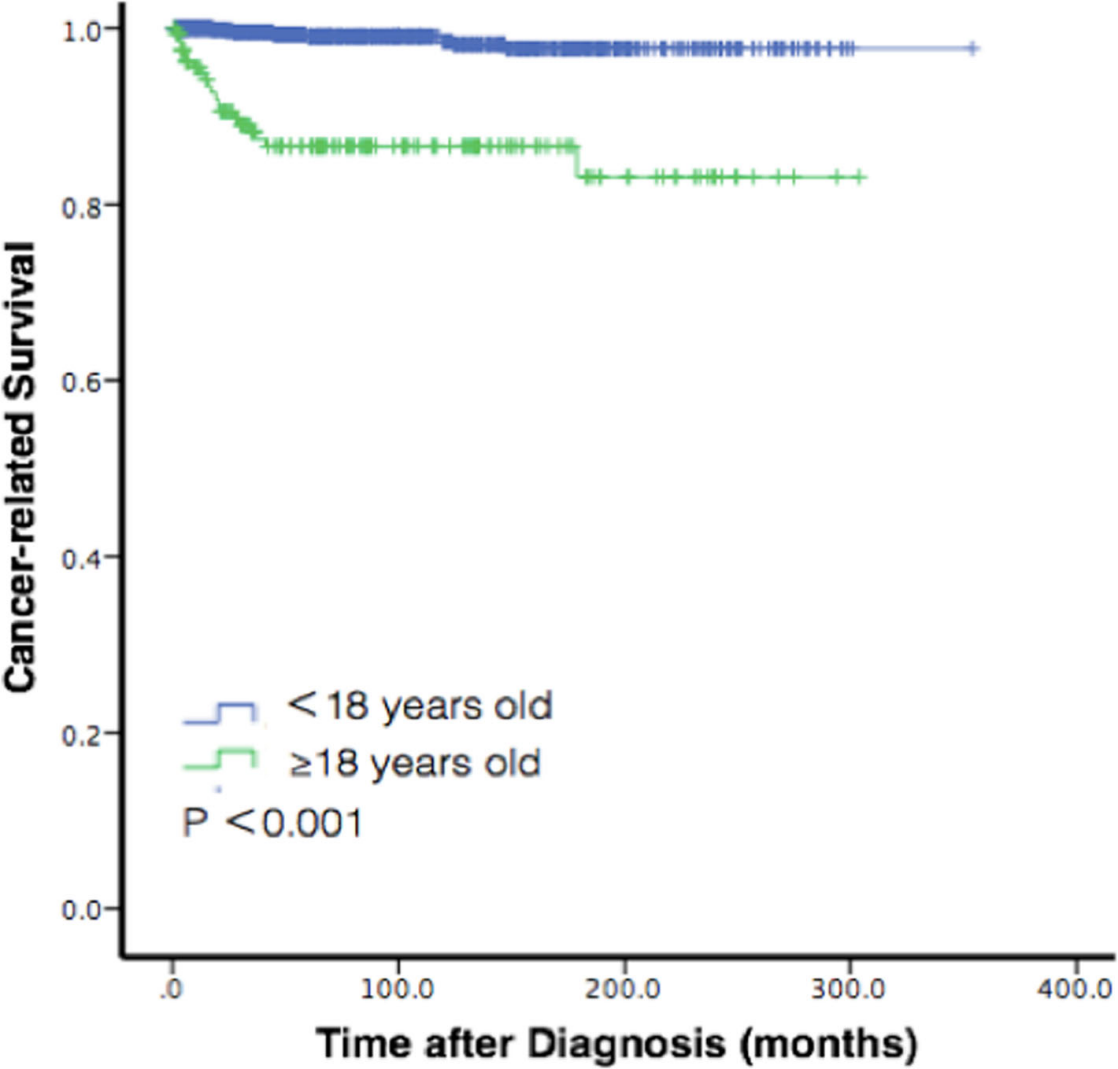
Kaplan-Meier survival analysis for cancer-related survival by age groups 0–18 and 18+ years

The OS at 1 year, 2 years, and 5 years after diagnosis was 99.2, 98.3, and 97.1%, respectively. The mean time of OS was 340.7 (95% CI: 335.9–345.6) months. In univariate analyses, sex (*P* = 0.186), race (*P* = 0.633), year of diagnosis (*P* = 0.279), tumor size (*P* = 0.078), tumor extension (*P* = 0.490), and laterality (*P* = 0.127) were not correlated with OS by Kaplan-Meier log-rank testing. Age (Fig. 1, Fig. 2), surgery performance, tumor grade, diagnostic confirmation, and histology type were statistically significant with OS (*P* < 0.001, respectively). In the multivariate analyses, age (P < 0.001, hazard ratio (HR) = 8.830, 95% CI: 4.088–19.073), tumor grade (P < 0.001, HR = 1.927, 95% CI: 1.516–2.450), diagnostic confirmation (P < 0.001, HR = 2.444, 95% CI: 1.632–3.660), and histology type (*P* = 0.046, HR = 1.563, 95% CI: 1.008–2.424) were significantly related with a decreased rate of survival after adjusting for sex, race, year of diagnosis, tumor size, tumor extension, and laterality. Patients who received surgery performance lived longer compared to those who did not, as it (HR = 5.501, 95% CI:2.664–11.358, p < 0.001) was associated with improved survival rate. We found surgery performance had a statistical effect on survival rate in univariate analysis, however, this phenomenon wasn’t found in multivariate analysis (when all factors were included) (Table 3).

**Table 3:** Univariate and multivariate analysis of tumor-related survival

	Univariate Analysis			Multivariate Analysis		
Variables	HR	95% CI	***p*** Value	HR	95% CI	***p*** Value
**Age(≥18, <18)**	11.289	5.284–24.122	<0.001	8.83	4.088–19.073	**<**0.001
**Sex(male vs female)**	0.606	0.288–1.274	0.186		/	
**Race**	1.135	0.675–1.908	0.633		/	
**Year of diagnosis(<2010, ≥2010)**	0.58	0.216–1.556	0.279		/	
**Surgery**	5.501	2.664–11.358	**<**0.001	1.452	0.664–3.173	0.35
**Tumor size**	4.115	0.854–19.815	0.078		/	
**Tumor grade**	2.88	2.332–3.557	**<**0.001	1.927	1.516–2.450	**<**0.001
**Diagnostic Confirmation**	2.436	1.803–3.292	**<**0.001	2.444	1.632–3.660	**<**0.001
**Tumor Extension**	1.157	0.764–1.752	0.490		/	
**Laterality**	1.296	0.929–1.808	0.127		/	
**Histology**	2.654	1.745–4.037	**<**0.001	1.563	1.008–2.424	0.046

## Discussion

To get a further knowledge of ONAs in patients, we conducted a retrospective study using the SEER program to evaluate the relation of various variables to ONAs. To the best of our knowledge, this current study is the largest retrospective study on ONAs. In this report, we identified 1004 cases with ONAs, diagnosed between 1984 and 2016. The results revealed a significant increase in the overall and ONAs-specific survival of ONAs of patients who were younger than 18 years old. Addition to age, tumor grade, diagnostic confirmation, and histology type were also independent prognostic factors in this patient population.

This study found that time of diagnosis was significantly related with survival rate. This study classified patients by age: younger thanand older than 18 years. Kaplan-Meier curves showed that the younger patients had better survival rate than the older groups, and 1-year survival rates for the 2 groups were 99.9 and 94.8%, respectively. The 5-year OS rates for both cohorts were 99.1 and 86.6%. Tumor formation had a less aggressive progress in infants than those in older people. A previous study suggested that a more advantageous genetic background involved with tumor formation of adolescents helped increased the survival of young patients compared with older cohort [32–34]. Thus, the underlying mechanism of tumor formation in children should have further investigation, then patients of different ages and biomarkers can receive appropriate treatment.

The study also showed a statistically significant increase in ONAs incidence rate in black patients who were older than 18 years old. Even though we did not find many previous studies about the relationship between the race and the incidence rate of ONAs specifically, previous researches on race and incidence of intracranial neurological tumors had found that black population had higher incidence rate and worse survival of numerous malignant tumors [35, 36]. Many research papers tried to explain the combination between the race and the incidence of neurological tumors, a previous article hinted that brain tumors might have different pathological classification between different races [37, 38].

The study illustrated an excellent OS at 5 years after diagnosis, which is 97.1% for the whole group. However, patients diagnosed with low-grade astrocytic tumors had a 5 year OS of 98.0%, while the 5 year OS of patients with high-grade astrocytic tumors was 34.3%. Thus, the different tumor histology type can explain the different clinical course of ONAs to a certain extent. The results of this study revealed that age of diagnosis was highly associated with tumor histology type, namely younger patients had a higher chance of being diagnosed with low-grade astrocytic tumors.

According to the information of the SEER registry, most of the patients with ONAs in US were diagnosed upon radiographic testing. Of all the 1004 patients diagnosed with ONAs, 67.1% of the whole population was diagnosed by radiography, and only 28.0% patients were diagnosed according to positive histology. The results were not unexpected to get since ONAs were tumors that were essential to the axons of the optic nerve, thus biopsy was not easily to carry out [38]. This may explain the reason why patients in the SEER program with ONAs had more chances of radiography imaging than histology, and these findings were consistent with other previous papers, whose results showed that the majority of patients were diagnosed on the basis of radiography imaging [39–41].

The limitations of this study were as follows, and lack of complete and comprehensive data collection within the SEER database should be the main reason. Even though we selected tumors located within the optic nerve, the exact locations were not recorded. A major limitation is the lack of information regarding visual outcomes. In clinic, visual outcome should be an endpoint with great importance for this disease, since the patients have an excellent overall survival. Therefore, the difference of the prognostic criterion may lead to the bias for the results. While surgery treatment is documented in the SEER information, it does not record the treatment with radiation treatment and chemotherapy in the SEER database. Chemotherapy is now considered as a prior treatment among all the choices for patients diagnosed with optic pathway gliomas, since it can delay or reduce radiation treatment [18, 42, 43]. Additionally, SEER program did not have information about tumor recurrence. A previous study showed that the complication of hydrocephalus was negative associated to survival rates in patients with astrocytoma [44]. However, our research couldn’t evaluate the association of hydrocephalus to the patients since this outcome was not kept in the SEER database. Last but not least, there may be selection bias when information recorded in medical centers.

The SEER database dated back more than 30 years, and therapeutic plan for ONAs had developed during that period. Nowadays, surgery is considered as the first-line treatment for ONAs and radiation and chemotherapy also have changed over time [32, 45, 46]. Patients recorded in the study may have received accordingly different treatments due to different preferences.

## Conclusions

This retrospective study of ONAs showed perspectives about variables that may affect survival rate. Younger age of patients seemed to be a protective factor, while patients with high-grade astrocytic tumors had a worse prognosis. Diagnostic confirmation and tumor grade were also independent prognostic factors in this patient population. More investigation is needed to look into this disease and try to find an excellent way to cure patients with this rare tumor.

## Data Availability

The dataset from SEER database generated and/or analyzed during the current study are available in the SEER dataset repository (https://seer.cancer.gov/).
